# Effect of Physiotherapy on an Elderly Patient With Distal Anterior Cerebral Artery Aneurysm Clipping

**DOI:** 10.7759/cureus.57225

**Published:** 2024-03-29

**Authors:** Maitri V Thamke, Snehal Samal, Bhumala P Vaidya

**Affiliations:** 1 Physiotherapy, Ravi Nair Physiotherapy College, Datta Meghe Institute of Higher Education and Research, Wardha, IND; 2 Neurophysiotherapy, Ravi Nair Physiotherapy College, Datta Meghe Institute of Higher Education and Research, Wardha, IND; 3 Neurological Physical Therapy, Ravi Nair Physiotherapy College, Datta Meghe Institute of Higher Education and Research, Wardha, IND

**Keywords:** subdural hematoma, physiotherapy, daca aneurysm clipping, daca aneurysm, distal anterior cerebral artery

## Abstract

This case study analyzes the effectiveness of physiotherapy in the treatment of a female patient, aged 65 years, with hypertension who had a history of subdural hematoma (SDH) secondary to a distal anterior cerebral artery (DACA) aneurysm rupture. For that, she underwent DACA aneurysm clipping surgery and developed a disability on the right side of her body. The patient had diminished functional independence, weak muscles, and restricted mobility when she first arrived. A thorough physiotherapy program was developed with the goals of increasing mobility, building independence in daily living tasks, and improving motor function. Adapted to the patient's particular requirements and limits, the intervention included therapeutic exercises, gait training, balancing exercises, and functional training. Assessments were carried out on a regular basis to track improvement and modify the treatment plan as necessary. The patient's motor function, mobility, and functional independence were significantly improved during the intervention. Physiotherapy played a crucial role in significantly improving the patient's recovery, quality of life, and right-side disability following the DACA aneurysm clipping surgery. The presented case study clearly highlights the value of early and focused physiotherapy intervention in the effective management of neurological impairments and in achieving better rehabilitation outcomes in patients with similar medical presentations.

## Introduction

Distal anterior cerebral artery (DACA) aneurysms account for 3-5% of all intracranial aneurysms, although they are extremely uncommon [1]. The prognosis for these aneurysms is known to be poorer than that of aneurysms in other places. The death rate for DACA aneurysms was 81.2% in cases where patients did not receive surgical treatment [2]. Even in patients who received surgical treatment, the rates of death and morbidity were 32% and 42.3%, respectively [3]. DACA aneurysms are present with surgical challenges because of their narrow working space in the interhemispheric fissure, dense adhesion between the cingulate gyri, parent artery control issues, broad-based and/or sclerotic necks, and frequent correlations with other vascular anomalies and multiple aneurysms [4]. First-degree intracranial aneurysms run in the family [5]. Cigarette smoking, heavy drinking, female gender, and hypertension are significant warning signs for cerebral aneurysm formation [6,7,8]. Aneurysms can manifest in several ways, such as headaches [9], unilateral third nerve palsy (posterior communicating artery aneurysm) [10], facial or orbital pain, epistaxis, progressive vision loss, and/or ophthalmoplegia (intercavernous internal carotid artery) [11], and signs of brainstem dysfunction (posterior circulation aneurysms). When direct, opposing forces rupture the thin walls of veins, blood is released inside the dura mater, causing a subdural hematoma [12]. Headache, psychological signs, seizures, cognitive impairment, and focal neurologic impairments are some of the symptoms of SDH [13]. SDH may also occur from ruptured aneurysms. All these conditions are caused by both lower-extremity weakness and frontal hemianopsia [14]. In this case, the DACA aneurysm rupture is the cause of the SDH. The aim of this study is to evaluate the effect of physiotherapy treatment on an elderly patient with DACA clipping surgery.

## Case presentation

A 65-year-old woman visited the outpatient department (OPD) complaining of vomiting and a strong headache that has persisted for the last five days. The vomiting has been persistent, occurring multiple times since its onset. She described the headache as severe, constant, and throbbing in nature. During the OPD visit, she rates her pain as a 10 out of 10, with no relief from analgesics. The headache has not been relieved with rest or changes in position. She denied any recent head trauma, unconsciousness, or seizures preceding the onset of symptoms. There was no history of fever, neck stiffness, or rash suggestive of infectious aetiology. Regarding her medical history, she has a known history of hypertension for the past two years, for which she takes antihypertensive medications regularly, but in the past few weeks, she started taking antihypertensive medications on alternate days. She denied any history of migraines or similar headaches in the past. On evaluation, the patient was conscious and drowsy, with a Glasgow Coma Score (GCS) of E3V4M6. After the initial resuscitation, the BP was 220/110 mmHg, which indicated an elevation in blood pressure. With this history, the patient went to the hospital on January 12, 2024, and after being examined, she was diagnosed with subdural hematoma on January 15, 2024. As a consequence of the distal ACA clipping surgery, the patient has developed a deformity on the right side of her body. The physiotherapy examination done on January 18, 2024, and the evaluation of joint reflexes are indicated in Table 1. The evaluation of muscle tone using a tone-grading scale is noted in Table 2.

**Table 1:** Evaluation of joint reflexes Ab, absent; +, diminished reflex response; ++, normal reflex response

Reflexes	Right side	Left side
Biceps jerk	Ab	+
Triceps jerk	+	+
Knee jerk	Ab	+
Ankle jerk	+	+

**Table 2:** Pre-intervention muscle tone according to TGS 1+ is decreased tone; 2+ is normal tone; 3+ is increased tone according to the tone grading scale (TGS)

Joints	Muscles	Right side	Left side
Shoulder	Flexors	1+	2+
	Extensors	1+	2+
	Abductors	1+	2+
	Adductors	1+	2+
	Internal rotators	1+	2+
	External rotators	1+	2+
Elbow	Flexors	1+	2+
	Extensors	1+	2+
Hip	Flexors	1+	2+
	Extensors	1+	2+
	Abductor	1+	2+
	Adductor	1+	2+
	Internal rotators	1+	2+
	External rotators	1+	2+
Knee	Flexors	1+	2+
	Extensors	1+	2+
Ankle	Dorsiflexors	1+	2+
	Plantar flexors	1+	2+

Investigations

**Figure 1:**
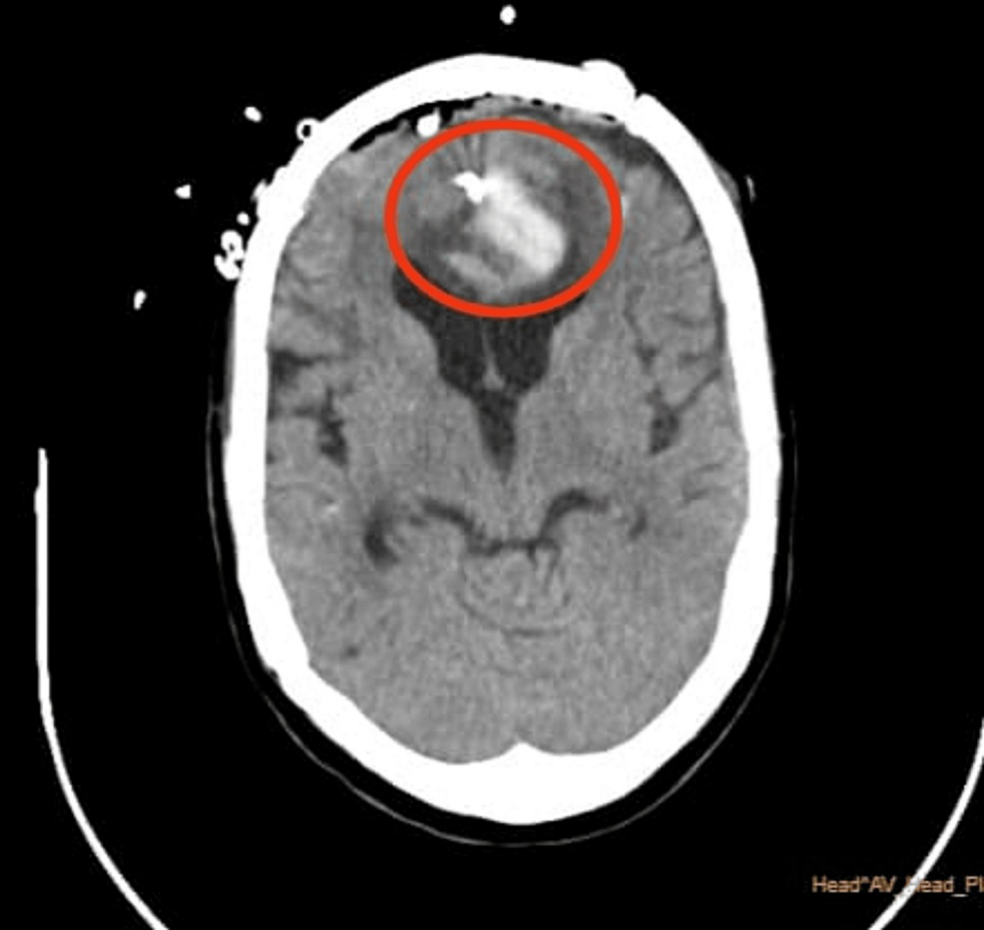
Preoperative computed tomography scan. The red circle shows the DACA aneurysm. DACA: distal anterior cerebral artery

**Figure 2:**
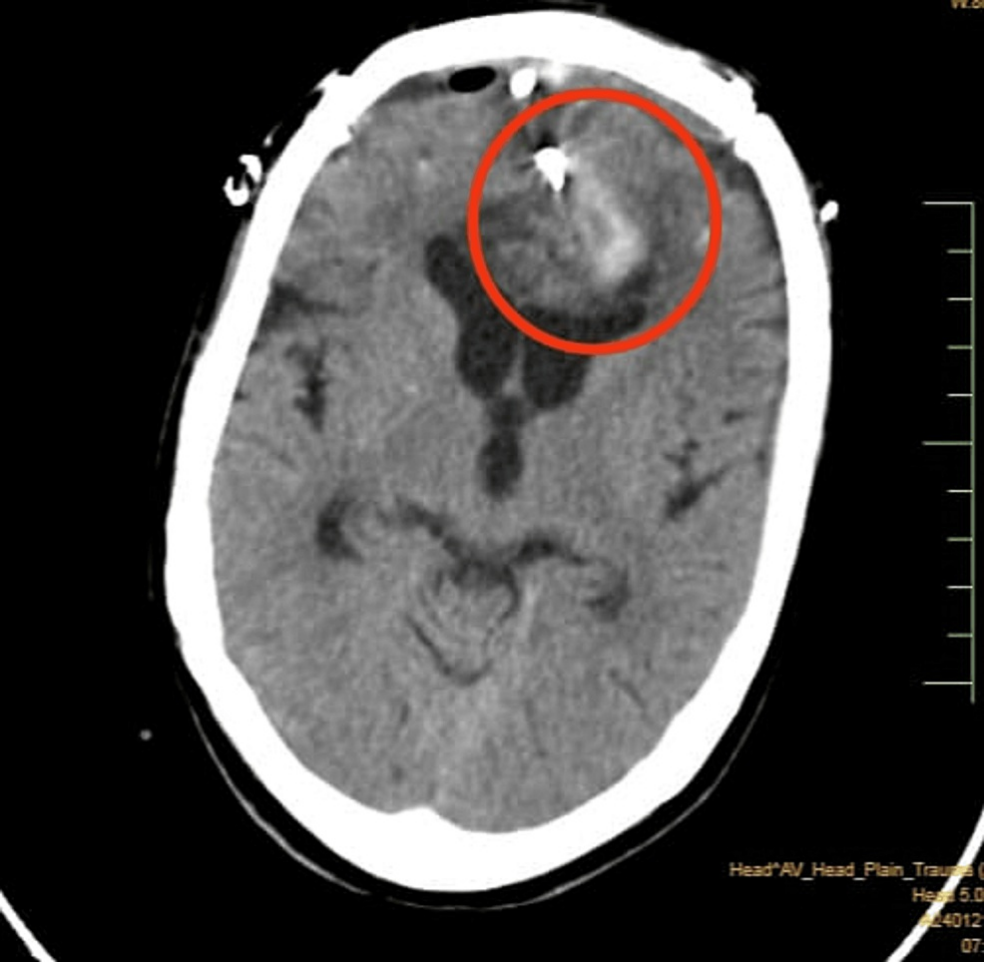
Post-surgery A CT brain shows the plaque calvaria defect.

On an MRI scan (plain and contrast), a large 27 x 26 mm T2/FLAIR hypointense and T1 mildly hyperintense lesion with blooming on gradient echo (GRE) sequences was seen in the left frontal parafalcine region. It showed a mild surrounding perilesional edema with effacement of the frontal horn of the left lateral ventricle. There was a visible extension of the hematoma along the falx, with intraventricular extension in both lateral ventricles. The CT brain plain before surgery is shown in Figure 1. In the post-surgery A CT brain (Figure 2), plaque calvaria defect was noted in the bilateral frontal region with overlying metallic clips, extra-calvarial swelling, air density foci, and drain tube, consistent with a postoperative status with an adjacent mixed-density subdural collection of a maximum thickness of 6 mm in the frontal region with air foci. Aneurysmal clips and air foci were seen in the anterior interhemispheric region. There was an intra-parenchymal hematoma in the inter-hemispheric region, involving bilateral frontal lobes more on the left; a mass effect was noted in the form of effacement of the frontal horn of the left lateral ventricle.

Physiotherapy intervention

**Figure 3:**
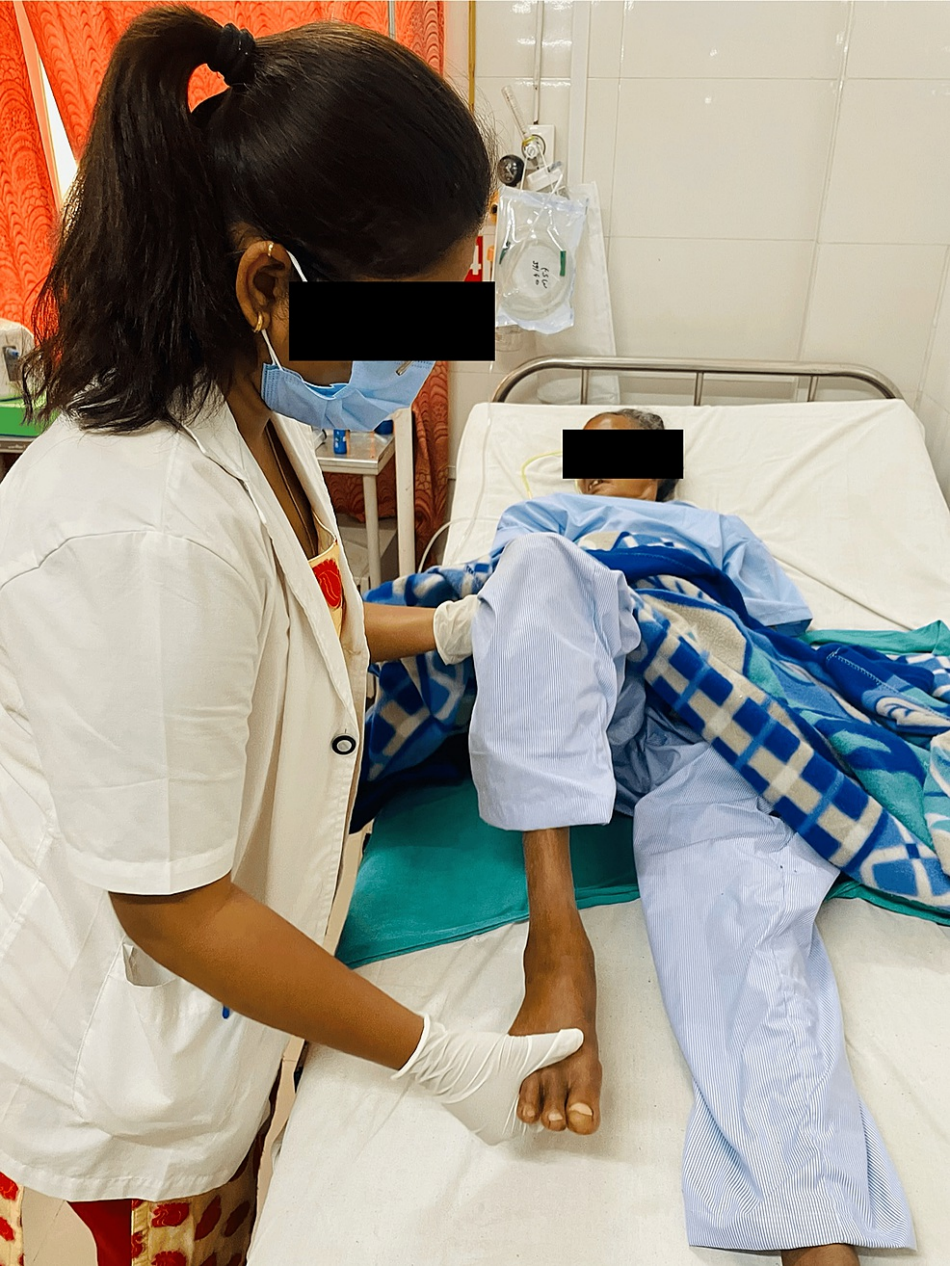
Movement of the lower limb passively (It refers to the manipulation of the leg joints without the patient actively contracting their muscles to produce the movement.)

**Figure 4:**
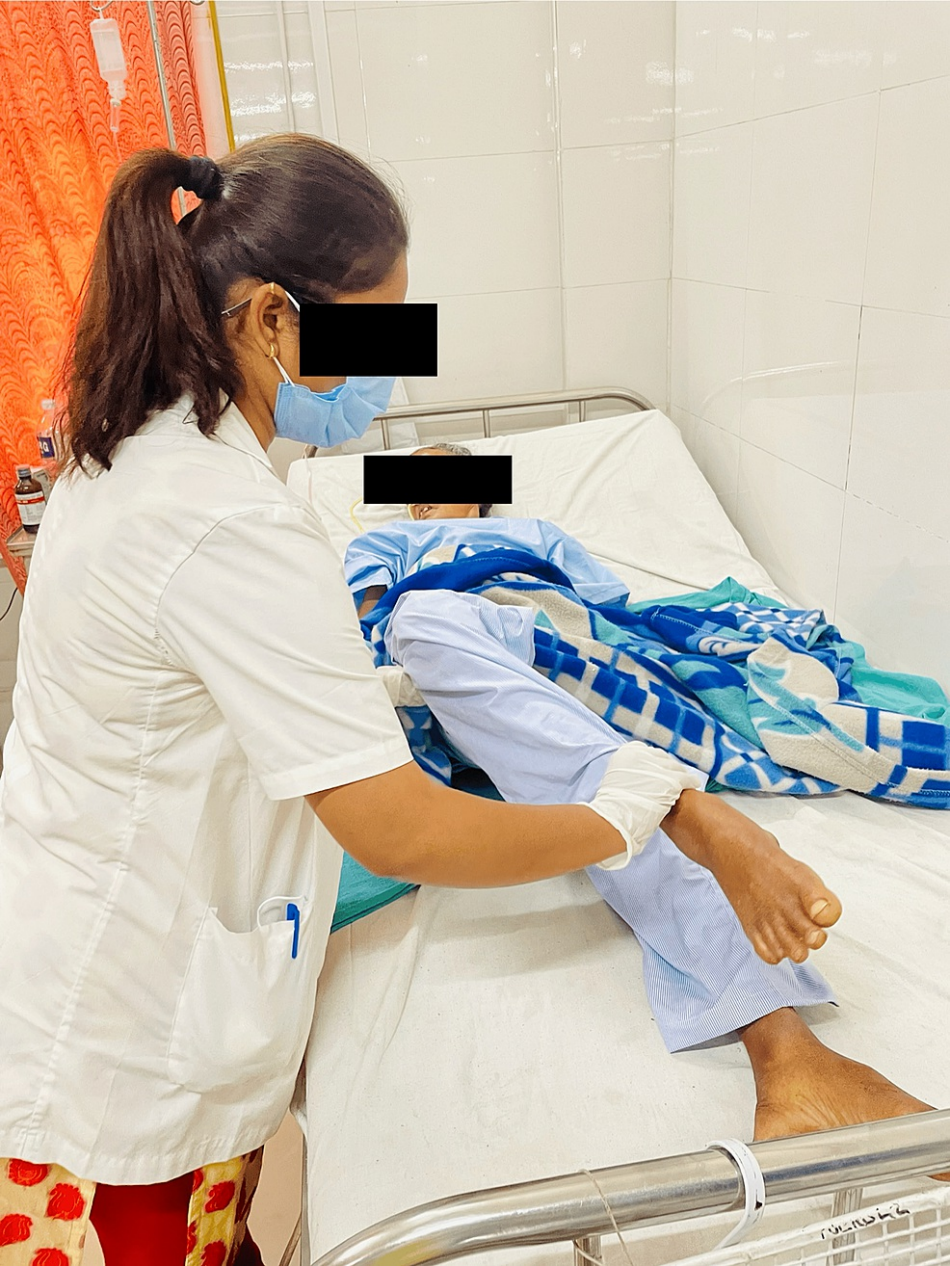
PNF lower extremities (PNF involves both passive and active movements to improve flexibility, strength, and neuromuscular control. ) PNF: proprioceptive neuromuscular facilitation

**Figure 5:**
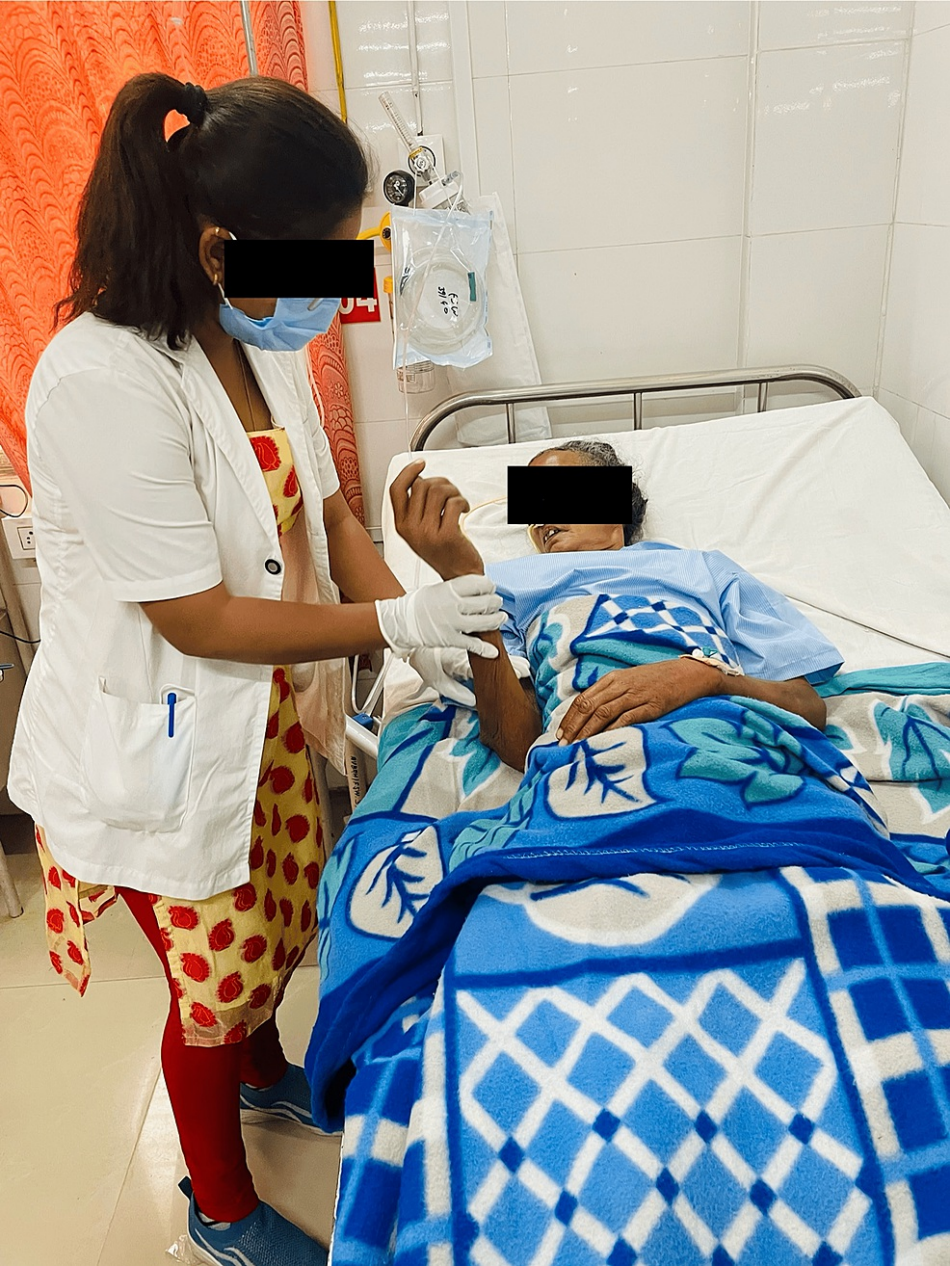
Rood's method: tapping (In order to promote muscle contraction or relaxation, improve muscle tone, and improve motor control, bicep muscles are tapped rhythmically, repeatedly, and under control)

A five-week physiotherapy rehabilitation treatment was given for one hour per day, five days each week. Table 3 provides an overview of the patient's physiotherapy intervention. The state of the patient's physical abilities, including muscle strength, balance, and coordination, were assessed after the patient was informed about the condition. The patient underwent bed mobility training and functional training to improve her ability to maintain balance and transfer. This training involved positioning techniques and learning proper body mechanics. Deteriorating health issues including balance issues, muscular weakness, incoordination, and cognitive dysfunction are caused by a number of circumstances [15]. The patient undergoing physical therapy is shown in Figures 3-5.

**Table 3:** Treatment plan given for five weeks PNF, proprioceptive neuromuscular facilitation; reps, repetitions

Goals	Intervention	Dosage
Patient education	To inform the patient's family about the specifics of the condition and the ways in which physical therapy can help	Patient counselling and regular patient updates
To restore muscle tone	Rhythmic start PNF flexion: Upper extremity (D1), lower extremity (D2) Roods method: tapping, rapid brushing, quick icing of the abdominal muscles, and intense joint compression	10 reps x 2 sets
To improve mobility of the upper and lower limbs	Movement of the left upper and lower limbs passively. Exercises for supported mobility that are passive for the wrist and elbow and active for the right upper and lower limbs	10 reps x 2 sets
To improvise deglutition and speech	Exercises for the lips and tongue (lip pursing, tongue protrusion, lateral tongue movement), oral sensory stimulation, and face and mouth workouts in front of a mirror	10 reps x 2 sets
To enhance bed mobility and transitions	Exercises for bed mobility include segmental rolling, logrolling, and supine-to-side laying	5 reps x 1 set
To increase balance and coordination	Perturbation and reach-out activities	10 reps x 2 sets
To prevent postural deformity	Position techniques	Every 2 hours
Functional training	Sit to stand, standing on different types of surfaces	5 reps x 2 sets

Outcome measures

Table 4 displays the results before and after therapy.

**Table 4:** Outcome measures

Outcome measures	On admission	At discharge
Functional independence measure scale	80/126	72/126
Modified Rankin scale	Grade 4	Grade 3

## Discussion

The results of this case study have been reviewed, showing that initial physiotherapy helped patients with cerebral aneurysms that had undergone surgical clipping to enhance their functional status with mobility of the upper and lower limbs, to improvise deglutition and speech and independence in daily living activities. For patients to reach complete independence following treatment for cerebral aneurysms, early physiotherapy is insufficient [16-18]. While microsurgical DACA aneurysm clipping is an established and reliable method with decent outcomes, its outcomes are not as good as those of anterior circulation aneurysm surgery. These days, endovascular therapy, which is growing rapidly, is just as successful in treating ruptured DACA aneurysms. DACA aneurysms are truly challenging to treat with surgery or endovascular techniques because of their fragility, small dome, large neck, widely distributed origin, and peripheral placement.

In the given article series, only 19 patients (67.8%) had favorable outcomes, six patients (21.5%) were severely disabled, and three patients (10.7%) died. The findings of the study are comparable to those of previously published series, but they fell short of expectations. This is likely because most published reports included a combination of ruptured and unruptured DACA aneurysms, whereas all of the patients in our cohort had burst aneurysms [19]. For this reason, rehabilitation needs to continue after discharge. The functional state and level of observation needed are affected by the severity of cognitive impairment [20]. The current case findings show that early mobilization is important to prevent complications. Strength training is given to increase muscle strength, range of motion exercises for flexibility of joints, and balance training for weight bearing; these are essential components of physiotherapy intervention. This report discusses pain management strategies and the significance of patient education and home exercise programs. It also advocates for a multidisciplinary approach and long-term management for optimal outcomes.

## Conclusions

This case study highlights the effectiveness of physiotherapy with comprehensive rehabilitation in improving the functional outcomes and quality of life for a patient who underwent DACA aneurysm clipping surgery and developed disability on the right side of her body. The patient showed remarkable improvement in motor function, mobility, and functional independence through a five-week physiotherapy program. The comprehensive physiotherapy intervention, including therapeutic exercises, gait training, balancing exercises, and functional training, was instrumental in addressing the patient's specific needs and limitations. Outcome measures evaluated the progression of the patient. Physical therapy plays a crucial role in preventing complications, enhancing functional activity, and promoting independence in daily living activities.
